# Enhanced Knowledge Distillation for Advanced Recognition of Chinese Herbal Medicine

**DOI:** 10.3390/s24051559

**Published:** 2024-02-28

**Authors:** Lu Zheng, Wenhan Long, Junchao Yi, Lu Liu, Ke Xu

**Affiliations:** 1 College of Computer Science, South-Central Minzu University, Wuhan 430074, China; 2 Key Laboratory of Information Physics Integration and Intelligent Computing of National Ethnic Affairs Commission, Wuhan 430074, China; 3 Hubei Provincial Engineering Research Center of Agricultural Blockchain and Intelligent Management, Wuhan 430074, China; 4 School of Computing and Mathematical Sciences, University of Leicester, Leicester LE1 7RH, UK

**Keywords:** Chinese herbal medicine, knowledge distillation, dual-teacher supervision, adaptive attenuation, portable application

## Abstract

The identification and classification of traditional Chinese herbal medicines demand significant time and expertise. We propose the dual-teacher supervised decay (DTSD) approach, an enhancement for Chinese herbal medicine recognition utilizing a refined knowledge distillation model. The DTSD method refines output soft labels, adapts attenuation parameters, and employs a dynamic combination loss in the teacher model. Implemented on the lightweight MobileNet_v3 network, the methodology is deployed successfully in a mobile application. Experimental results reveal that incorporating the exponential warmup learning rate reduction strategy during training optimizes the knowledge distillation model, achieving an average classification accuracy of 98.60% for 10 types of Chinese herbal medicine images. The model boasts an average detection time of 0.0172 s per image, with a compressed size of 10 MB. Comparative experiments demonstrate the superior performance of our refined model over DenseNet121, ResNet50_vd, Xception65, and EfficientNetB1. This refined model not only introduces an approach to Chinese herbal medicine image recognition but also provides a practical solution for lightweight models in mobile applications.

## 1. Introduction

Chinese herbal medicine stands as a distinctive therapeutic approach within traditional Chinese medicine (TCM), offering a diverse range of remedies for various ailments. However, the expansive array of Chinese herbal medicines used across different regions has given rise to a concerning trend: the proliferation of counterfeit and substandard substitutes on the market. This poses significant risks as ordinary consumers, lacking in-depth knowledge, often inadvertently consume these falsified products. The complexity of Chinese herbal medicine compounds this issue, making it challenging for laypersons to accurately identify genuine herbs. As a consequence, mistaken ingestion remains a frequent occurrence among consumers. Presently, the identification and classification of these herbs heavily rely on individuals with specialized expertise in this field. To address these challenges, the integration of deep learning technology into the recognition and classification of Chinese herbal medicine becomes imperative. The remarkable advancements in image recognition offered by deep learning present a promising solution. This integration holds immense potential in revolutionizing traditional Chinese medicine (TCM) by providing a systematic approach to identifying and authenticating herbal medicines. The application of deep learning in research aimed at recognizing and categorizing Chinese herbal medicine marks a crucial step forward in preserving and advancing the legacy of TCM. Its incorporation promises to empower both practitioners and consumers by enhancing the authentication and classification of these invaluable remedies.

In recent studies, deep learning technologies have been harnessed for advancing Chinese herbal medicine identification. Huang et al. [1] proposed a Chinese herb image classification method based on AlexNet. Through meticulous data augmentation and parameter fine-tuning, they achieved an impressive classification accuracy of 87.5% after 300 epochs. Gao et al. [2] introduced a recognition approach for natural grassland plant species using Inception_V3 with Tensorflow, achieving a peak accuracy of 89.41% in the model’s validation dataset’s Top1 error. Zhang et al. [3] contributed by classifying 17 types of Chinese herbs through a VGG network, attaining an outstanding average recognition accuracy of 96% in the validation dataset. Their model was further deployed on mobile devices, demonstrating practical application. Wang et al. [4] proposed an image recognition method for Chinese herbal plants based on the AlexNet network. Utilizing a deep coding and decoding network, they successfully trained the model to classify 15 types of Chinese herbal images, achieving an impressive average classification accuracy of 99.38%, but the large model parameter amount led to large training and inference computation, requiring more memory and computing resources, which was not suitable for mobile deployment. Hu et al. [5] introduced a dual-channel U-shaped convolutional neural network with feature calibration. They generated a training model for single-view fritillaria image data, surpassing the classification results of traditional machine learning methods. Moreover, by incorporating multi-view fritillaria images and employing a three-dimensional convolutional neural network, they developed a more precise fritillaria classification model. While these scholars have conducted profound research in Chinese herbal medicine image recognition, there is still room for improvement in model accuracy. An improved model has to not only maintain high classification accuracy but also focus on reducing the model parameter amount to improve performance. Moreover, concerns arise regarding the redundancy of model parameters, hindering deployment on mobile devices due to inadequate detection speed.

To address the aforementioned challenges, this paper proposes an approach called dual-teacher supervised decay (DTSD) for adaptive-decay knowledge distillation, which aims to enhance the performance of the standard model. By enhancing the output soft label, adapting decay parameters, and dynamically combining loss functions from the teacher model, DTSD is employed in the MobileNet_v3_Small network to enable accurate predictions despite its smaller size. Consequently, the accuracy of the MobileNet_v3_Small network is elevated to match that of more complex networks. The proposed model is then integrated into an intelligent Chinese herbal medicine recognition system for mobile devices, facilitating the efficient recognition and classification of Chinese herbal medicine.

The main contributions of our work include:

1. Proposing a dual-teacher supervised model to reorganize the predictive distribution of dual teachers to achieve more accurate and robust soft labeling, which improves the performance of the model.
2. Dynamically adjusting the temperature parameter T and the weight distribution value λ between the teacher model and the real label to gradually reduce the influence of the teacher model in the training process, so that the student model can more flexibly balance the complexity and the model’s generalization ability in the training process.
3. Adopting JS scatter with symmetry to replace the cross-entropy loss of the predicted values of the soft label and the student model to better capture the similarity between the distributions and prompt the student model to better inherit the knowledge of the tutor model.
4. A lightweight MobileNet_v3 network-based herbal medicine recognition system is implemented, and by applying our proposed DTSD method to the MobileNet_v3_Small network, we improve the accuracy and robustness of herbal medicine recognition while maintaining a small model size.

The rest of this paper is organized as follows: The preliminaries and dataset collection are presented in the first half of Section 2. Also in that section, the dataset augmentation strategies are described. Then, we introduce the knowledge distillation model with dual-teacher supervised decay in the second half of Section 2. Comparative experiment results and analysis are given in Section 3 to verify the effectiveness of the proposed methods, followed by a short conclusion in Section 4.

## 2. Materials and Methods

### 2.1. Dataset Collection

**Figure 1:**
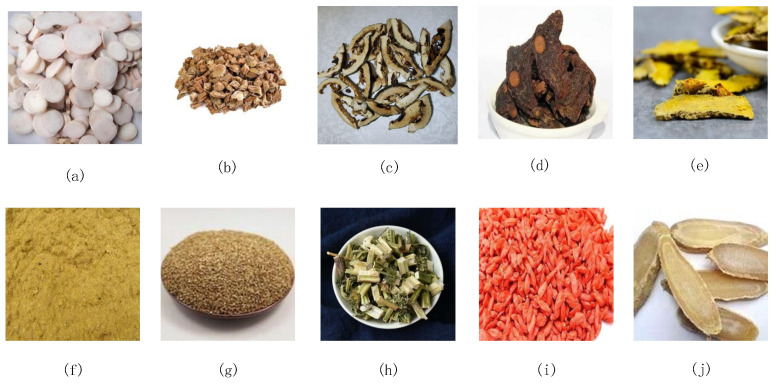
Sample of the Chinese herbal medicine dataset: (**a**) Radix paeoniae; (**b**) Radix stemonae; (**c**) Fructus aurantia tablets; (**d**) Polygonatum; (**e**) turmeric; (**f**) Pollen typhae; (**g**) Cnidium monnieri; (**h**) motherwort; (**i**) Chinese wolfberry; (**j**) curcuma.

The dataset utilized in this study consists of images depicting 10 distinct types of Chinese herbal medicine. These images were gathered from the Internet using a Web crawling technique and subsequently underwent a meticulous process of curation and filtration. A Web crawler first needs to determine the initial URL to be crawled and then builds a queue of URLs by parsing links on the page. The crawler accesses the web pages one by one according to the URLs in the queue. The page is requested from the server via an HTTP request, and then the HTML data returned by the server are downloaded locally. The downloaded pages are usually in HTML format, and the crawler needs to parse the HTML to extract useful information. The parsed data were the herbal images. This process resulted in a total of 1000 images of Chinese herbal medicines being compiled. The dataset, as illustrated in Figure 1, encompassed 10 specific types of Chinese herbal medicines, namely *Radix paeoniaealba*, *Radix stemoonae*, *Fructus aurantia tablets*, *Polygon atum*, *turmeric*, *Pollen typhae*, *Cnidium monnieri*, *motherwort*, *Chinese wolfberry*, and *curcuma*. By cropping and compression, each category comprised 100 images, all of which sized at 320 pixels by 320 pixels and possessing a resolution of 96 dots per inch. During the training of the teacher network, the dataset was partitioned into a 7:2:1 ratio, with 700 images (70%) allocated to the training dataset, 200 images (20%) to the validation dataset, and 100 images (10%) to the test dataset. The dataset was divided into datasets according to the 7:2:1 ratio for each category. The authors labeled various types of herbs.

### 2.2. Data Enhancement

**Figure 2:**
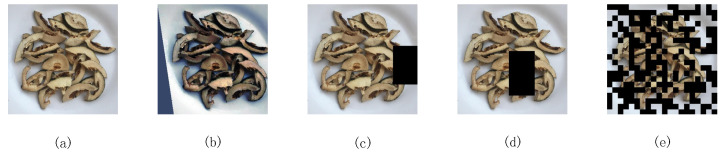
Data augmentation: (**a**) original image; (**b**) random augmentation strategy; (**c**) cutout strategy; (**d**) random erasing strategy; (**e**) hide-and-seek strategy.

The performance and recognition ability of the model during training are influenced by the generalization and quantity of data. When the available data are limited, overfitting becomes a more prominent issue in deep learning models. To address this challenge and improve the model’s generalization capabilities, data augmentation techniques are employed prior to training. These techniques enable the generation of more diverse data representations, as depicted in Figure 2. In order to preserve the original data features, the dataset was expanded to a size of 10,000 through generalization under simulated real conditions. The augmentation strategies [6] primarily included an image transformation class and an image cropping class.

#### 2.2.1. Image Transformation Class

For the rand augmentation strategy, specific probability distributions were set for each sub-strategy. The transformations included rotation (±30 degrees), flipping (50% probability of a horizontal flip), cropping (up to 20% crop of the original image size), contrast adjustment (±10%), and other sub-strategies. Each sub-strategy was randomly applied with a uniform probability distribution, reducing the need for manual selection. Moreover, all sub-strategies were also applied with equal probabilities, resulting in multiple augmentation sub-strategies being concurrently applied to a single image through probability combination. This approach allowed for the adjustment of image brightness, contrast, saturation, and hue simultaneously, simulating variations in shooting angles and actual lighting conditions. The probability of applying each sub-strategy was set at 10%, ensuring a balanced augmentation without overpowering the original image characteristics. By incorporating random factors to mimic real-world lighting differences, the parameters aligned more closely with reality, reducing the impact of image angle and lighting variations, and enhancing the model’s robustness.

#### 2.2.2. Image Cropping

In the cropping class, cut out, random erasing, and hide-and-seek strategies were employed, each with a distinct probability of application: 15% for cut out, 10% for random erasing, and 5% for hide and seek. The main objective of these strategies is to imitate classification scenarios where the subject is partially occluded in real-world situations. The size of the cropped area ranged from 10% to 20% of the original image, randomly chosen for each application. This helped prevent the model from becoming overly sensitive to salient regions of the image, thus avoiding overfitting.

### 2.3. Teacher Model: ResNet_vd

ResNet, introduced by Kaiming [7], aimed primarily to reduce the computational expense during network training and address issues related to diminishing or amplifying gradients leading to performance degradation with increasing network depth. This architecture employs stacked nonlinear layers to accommodate skip connections, thereby establishing an identity mapping. This ensures that deeper layers perform as effectively as shallower networks [8].

**Figure 3:**
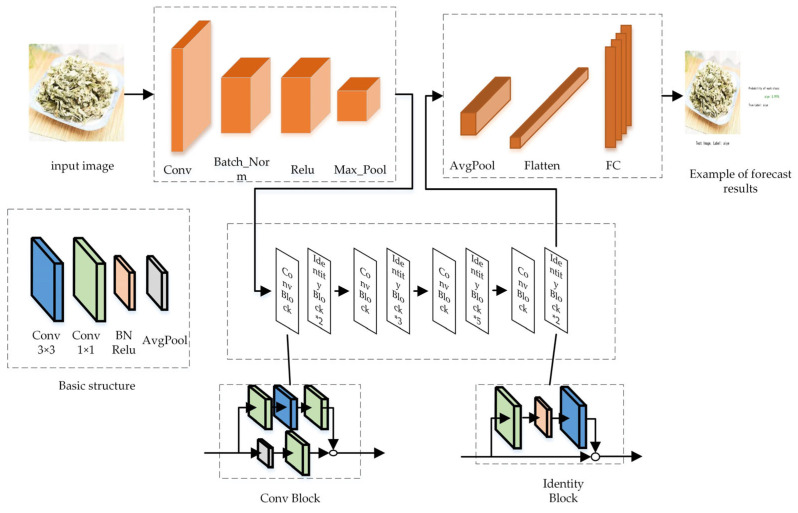
Resnet_vd model structure diagram.

The ResNet_vd model, an enhancement of ResNet by Tong et al., introduces various versions of residual modules [9]. Experiments conducted by He et al. demonstrate that ResNet_vd achieves significantly higher accuracy compared to other structural variations, leading to an approximate 0.85% increase in the top-1 accuracy on ImageNet. The network’s structure is illustrated in Figure 3.

### 2.4. Teacher Model: DenseNet

**Figure 4:**
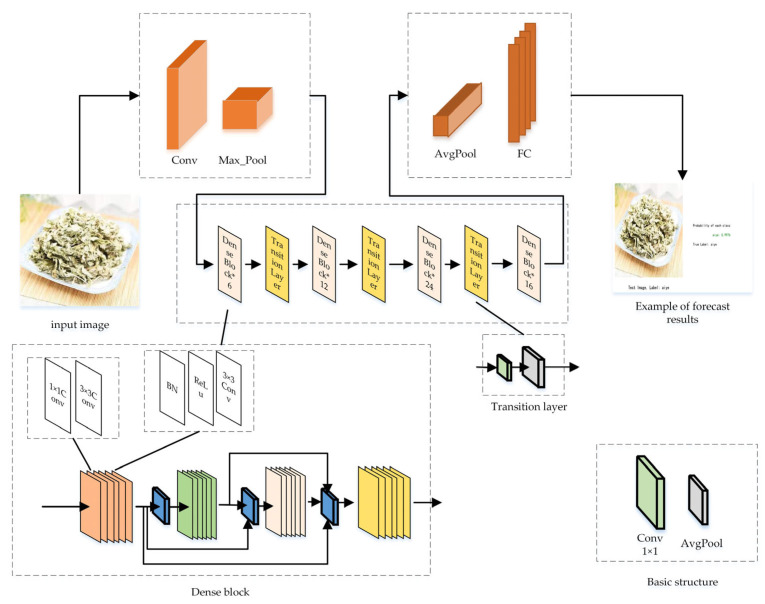
Densenet121 model structure diagram.

DenseNet, proposed by Gao [10] et al., emerged subsequent to an analysis of ResNets, highway networks, FractalNets, and other models. These authors highlighted a crucial attribute shared by these models: the construction of shortcuts between preceding and succeeding network layers, ensuring an identity mapping between them. Leveraging this characteristic, they developed an enhanced connection mode: each layer receives the feature maps from all preceding layers as input, as depicted in Figure 4. Research indicates that this connection method notably enhances the transfer of features and gradients within the network.

Compared to the residual network (ResNet), DenseNet achieves equivalent accuracy on the ImageNet dataset while utilizing less than half the number of parameters and computational resources [11]. Simultaneously, it demonstrates robust resistance to overfit-ting and displays strong generalization performance [12].

### 2.5. Student Model: MobileNet_v3_Small

In the realm of deep convolutional network models, achieving high accuracy often comes at the cost of increased model size and slower prediction speeds due to the incorporation of various techniques. The choice of MobileNet_v3 as the student model in our study over other lightweight models was motivated by its unique balance of efficiency and performance. Compared to other lightweight architectures, MobileNet_v3 offers an optimal trade-off between accuracy and speed, crucial for real-time applications on embedded devices. This balance is achieved through its advanced architectural innovations that reduce computational demand without significant loss in accuracy [13,14].

**Figure 5:**
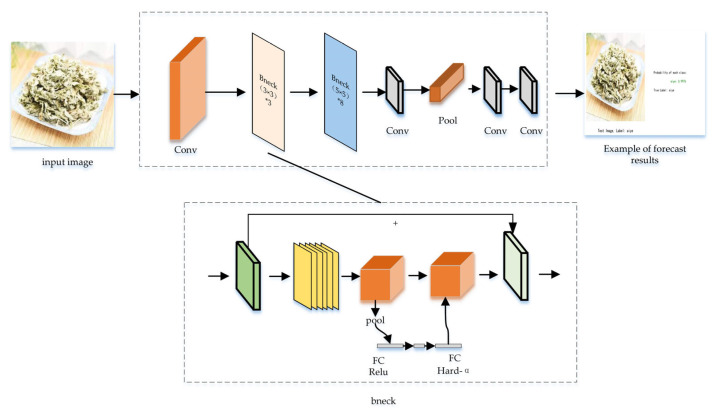
MobileNet_v3 model structure diagram.

MobileNet_v3 represents the next evolutionary step: a lightweight network that amalgamates the essence of MobileNet_v1 and MobileNet_v2 while introducing enhancements. It was selected for its superior efficiency in processing speed and reduced parameter count, critical for deployment in resource-constrained environments. This iteration revolves around four core blocks: (1) a depthwise-separable convolution; (2) an inverted residual structure with linear bottleneck; (3) a lightweight attention block; (4) the utilization of h-swish as an activation function, replacing the conventional swish [15,16,17,18]. The architecture of MobileNet_v3, depicted in Figure 5, demonstrates these key components.

### 2.6. Knowledge Distillation Model with Dual-Teacher Supervised Decay

**Figure 6:**
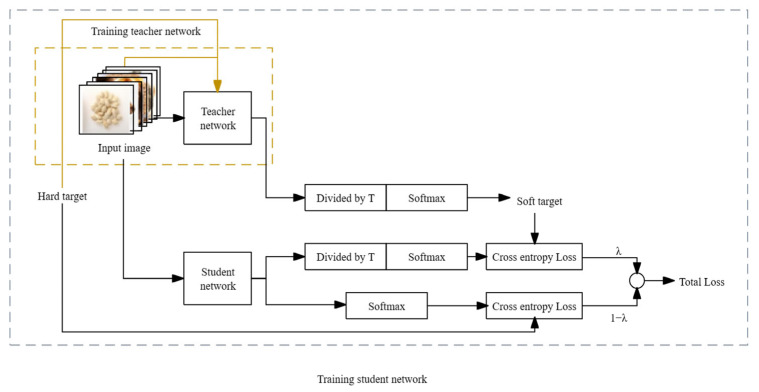
Flow chart of knowledge distillation.

In deep neural networks, the presence of a large number of parameters leads to redundancy. Knowledge distillation, as proposed by Hinton et al. [19], emerges as a technique to address this issue by compressing the model and reducing the parameter count [20,21,22,23,24]. The fundamental idea behind knowledge distillation involves incorporating soft labels associated with the teacher network into the total loss. This integration guides the training of the student network, facilitating knowledge transfer. The improvement in the performance of the student network is achieved while keeping the number of parameters constant. The resulting performance metrics closely align with those of the larger model. The detailed process is illustrated in Figure 6, and it unfolds as follows:

1. The teacher network initially trains on hard targets. Once the model is trained, just before the network performs softmax normalization on the output, each term is divided by a fixed temperature, T. This process yields the soft targets used to guide the learning of the student network.
2. During the training of the student network, the loss value employed for updating parameter weights during backpropagation is divided into two components. One part represents the cross-entropy loss computed on the true labels of the training dataset. The other part corresponds to the loss calculated on the soft output of the teacher network. Ultimately, these two losses are weighted and combined to generate the overall loss, which is then applied to the training of the student network model [25,26,27,28].

The standard knowledge distillation process has shown its innovative aspects, but it still faces the following issues, leading to a decrease in the accuracy of the student model compared to the general model in certain training scenarios:

1. Quality of guidance from the teacher model: Sometimes, the complex model might not predict perfectly. This is like a chef giving slightly incorrect cooking instructions to an apprentice. When these predictions, or guidance, are enhanced to make them more detailed for the student model (akin to increasing the “temperature” to make the lessons more intense), it can introduce errors or “noise”. This may lead the student model to learn incorrectly, like an apprentice learning flawed cooking techniques.
2. Adjusting the intensity of teaching (temperature): In past research, the intensity or detail in the teacher’s guidance was often set at a fixed level, usually moderate. But it is now understood that this should vary throughout the training, much like adjusting teaching methods for students as they progress. The “temperature”, or level of detail and complexity in the teacher’s guidance, needs to be adaptable, increasing or decreasing at different stages of the student model’s learning.
3. Balancing real data vs. teacher’s predictions (loss weighting λ): In traditional teaching methods, the balance between real-world data (hard labels) and the teacher’s predictions (soft labels) is constant. However, it is more effective if this balance changes over time. As the student model learns, the emphasis should gradually shift from what the teacher model predicts to what is actually observed in real-world data, allowing the student model to become more adept at handling real situations independently.

**Figure 7:**
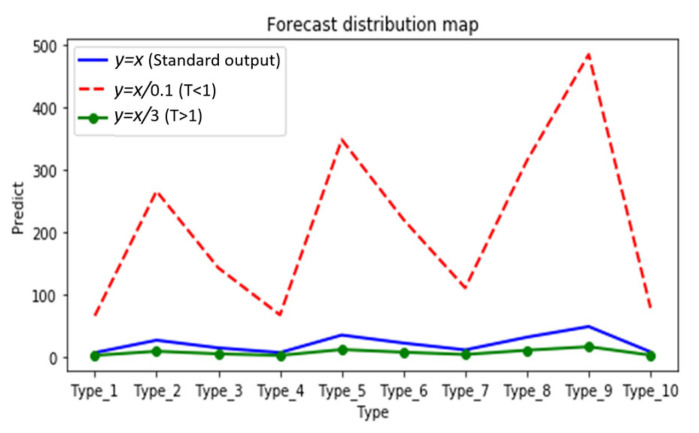
Distillation distribution of model output probabilities for different *T* cases.

**Figure 8:**
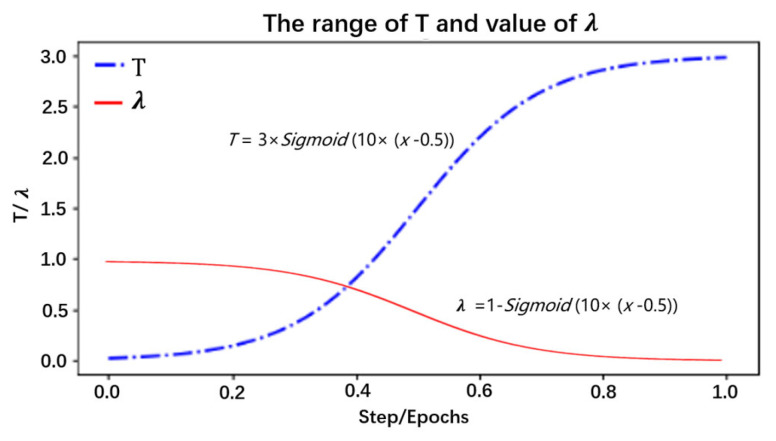
The distribution of *T*/*λ*.

Based on these issues, an improved model of adaptive-decay knowledge distillation with dual-teacher supervision is proposed, with specific improvements as follows:

1. The combination of soft labels: In standard knowledge distillation, we expand the teacher model from a single teacher to dual teachers to obtain multiple prediction distributions. To maintain the accuracy of the prediction distributions while acquiring more dark knowledge, we recombine the prediction distributions of the two teacher models. This is done by taking the maximum value of the predictions from the two teacher models in each dimension as the category classification result for that dimension, thereby obtaining a soft label with greater accuracy and richer dark knowledge. The formula is shown as Formula (1). Here, *p* and *q* represent the predicted labels given by the two teacher models, respectively. Through this formula, we generate a new probability distribution composed of the maximum values from two different probability distributions in each dimension.
  (1) Max_out(p,q)=[Max(p_1,q_1),Max(p_2,q_2),…Max(p_n,q_n)]
2. Selection of *T*: By analyzing the distillation distribution of the model output probability for different *T* cases, as shown in Figure 7, the different types are 10 classifications for herbal recognition, and the standard output is the blue solid line. When the value of *T* is smaller than 1 (the red dotted line), the gap between the true prediction value and the dark knowledge is enlarged, that is, the proportion of the true prediction increases. When the value of *T* is greater than 1 (green dotted line), the total prediction distribution is smoother, which means the proportion of dark knowledge is increased. Therefore, in the early stage of training, *T* is set to a value smaller than 1, so that the student model can quickly find the basic proper parameters in the early stage. With the deepening of training and the expansion of the proportion of dark knowledge, the student model with high accuracy further learns the dark knowledge part of the correct prediction distribution given by the teacher model, so as to improve its accuracy. Thus, the value of *T* is set to the value of the function that grows with the training epochs. As illustrated in Formula (2) *x* is the training metric; through this function, the temperature *T* changes with the *x* in an S-shaped curve and is defined as the deepening of the experiment (step/epochs), increasing in an S-shaped curve, and the main value range is [0–3], so that the student model can learn different degrees of dark knowledge in different epochs. This is shown in Figure 8. The student model in the early stage as a low weight; as the model training process continues to rise, the relationship is well reflected as a sigmoid function, that is, an “S” curve. We have adjusted the parameters of the sigmoid function so as to be more in line with the whole training process of the model.
  (2) $$T\_function\left(x\right)=3\times Sigmoid(10\times \left(x-0.5\right))$$
3. Selection of *λ*: In knowledge distillation, when the student model is at distinct training phases, the combined weights of the teacher model and the true label are likewise diverse. In the early stage of training, transfer learning and real labels are mainly mixed for learning and fitting, which guarantees that the high accuracy based on the pretrained model can be acquired in the whole model training. Nevertheless, with the deepening of the training epochs, since the student model has reached a successful convergence situation through self-study, the accuracy cannot be further improved. Therefore, by increasing the proportion of the teacher model on and on, the student model learns the dark knowledge distribution from the teacher model prediction distribution, thereby improving the model performance. The change in *λ* is shown in Figure 8 and Formula (3), where *x* indicates the training times. By this function, *λ* decreases in an S-curve with the deepening of the training process.
  (3) $$\lambda \_function\left(x\right)=1-Sigmoid(10\times (x-0.5))$$
4. Calculation of the loss:
  (a) In the loss calculation of the soft label and the student model, the Jensen–Shannon divergence with symmetry is utilized to replace the cross-entropy loss as the similarity measure metrics of two prediction distributions, as shown in Formula (5).
  (b) In view of the one-hot characteristic of the hard label, the loss between the hard label and the student model is still computed via the cross-entropy loss, as shown in Formula (6).
  (c) The total loss is derived from Formulas (7)–(12).
    (4) $$KL(P,Q)=\sum p(x)\log\frac{p(x)}{q(x)}$$
    (5) $$JS(P_{1},P_{2})=\frac{1}{2}KL(P_{1},\frac{P_{1}+P_{2}}{2})+\frac{1}{2}KL(P_{2},\frac{P_{1}+P_{2}}{2})$$
    (6) $$CE(Lable,Predict)=-\sum_{j=1}^{N}{Label}_{j}\cdot \log\left({Predict}_{j}\right)$$
    (7) T=T_function(step/epochs)
    (8) λ=λ_funciton(step/epochs)
    (9) $${Out}_{teacher}=Max\_out({Out}_{teacher1},{Out}_{teacher2})$$
    (10) $$L_{soft}=JS({Out}_{teacher}/T,{Out}_{student}/T)$$
    (11) $$L_{hard}=CE(Hard_{label},{Out}_{student})$$
    (12) $$Loss=\left(1-\lambda \right)*L_{soft}+\lambda *L_{hard}$$

In Formula (4), KL represents the relative entropy formula, *P* and *Q* represent two probability distributions, respectively, and *p*(*x*) and *q*(*x*) are the specific probabilities in a certain dimension. In Formula (5), *JS* is the loss function for calculating the two probability distributions, *P*_1_ and *P*_2_ are two distinct probability distributions, and the function returns the JS divergence loss between these two distributions. In Formula (6), *CE* is the cross-entropy function of two probability distributions, Label and Predict are usually the prediction probability distributions given by the true label and the student model, respectively. In Formulas (7)–(12), the above formulae are called. *T* represents the temperature metric during distillation, *λ* represents the combination weight between two different losses, where the quotient of step (current training number) and epochs (total training number) is used as the training progress index. Outx reflects the probability distribution given by model *x*. *L* represents the two losses computed by different calculation methods, and eventually they are merged by the *λ* weight to synthesize the final loss.

**Figure 9:**
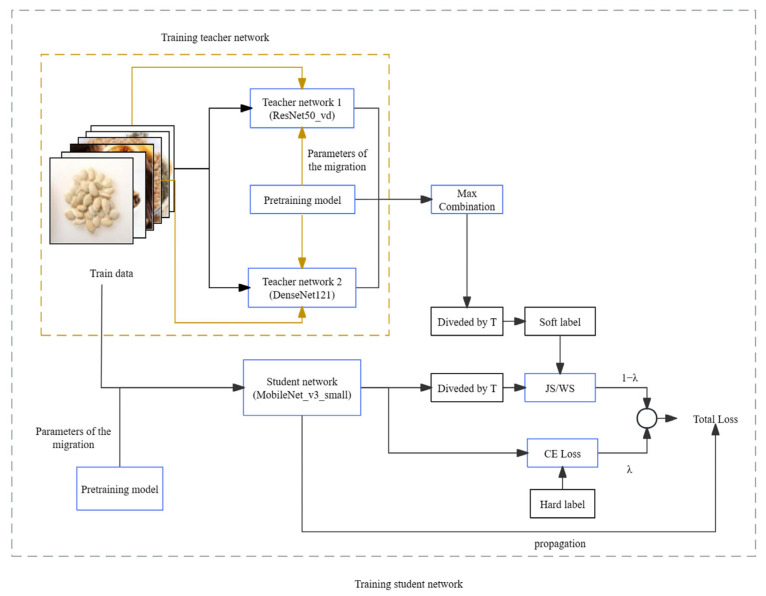
Flow chart of DTSD distillation.

The improved dual-teacher supervised decay (DTSD) has two teacher models selected as the complicated and high-accuracy models ResNet50_vd and DenseNet121, respectively. The student model chosen for this paper was MobileNet_v3_Small, which is known for its lightweight design. The model’s structure is depicted in Figure 9.

## 3. Results and Discussion

### 3.1. Experimental Setting

The primary experimental setup for this paper consisted of: (1) A desktop computer operating on Windows 10, equipped with an Intel Xeon E5-2630 v4 processor at 2.2 GHz, 64 GB of RAM, a 1.5 TB mechanical hard drive, and an NVIDIA Tesla P4 graphics card with 32 GB of video memory. This setup leveraged GPU acceleration for computations and was configured with the Paddle deep learning framework within a programming language environment. (2) Baidu’s open platform, Ai Studio, featuring an NVIDIA Tesla V100 graphics card, utilizing GPU computing power and acceleration, with the Paddle deep learning framework also implemented in a programming language environment.

### 3.2. Experimental Design

First, we pretrained the ResNet50_vd, DenseNet121, and MobileNet_v3_Small network models on the public dataset ImageNet2012 [29], mainly fine-tuning the models to verify that pretraining could improve the model performance. Subsequently, we trained the student model MobileNet_v3_Small under the guidance of the dual teacher models ResNet50_vd and DenseNet121, to validate the DTSD model and perform optimal parameter tuning. Furthermore, to verify the trained MobileNet_v3_Small_DTSD, we compared it with the similar MobileNet_v3_Small and other classic models in comparative experiments. To minimize the randomness of training, the data presented in the table are average values obtained from multiple measurements. Moreover, the dataset used was an augmented dataset of 10 types of Chinese herbal medicines.

### 3.3. Experiments on Boosting Training with Pretrained Models

To verify the impact of the pretrained models on the training process and the final model performance, comparative experiments were conducted for the two teacher models, ResNet50_vd and DenseNet121, as well as the student model MobileNet_v3_Small, using pretrained models. All the mentioned models were first pretrained on the public dataset ImageNet2012, followed by transfer learning on a Chinese herbal medicine dataset.

**Figure 10:**
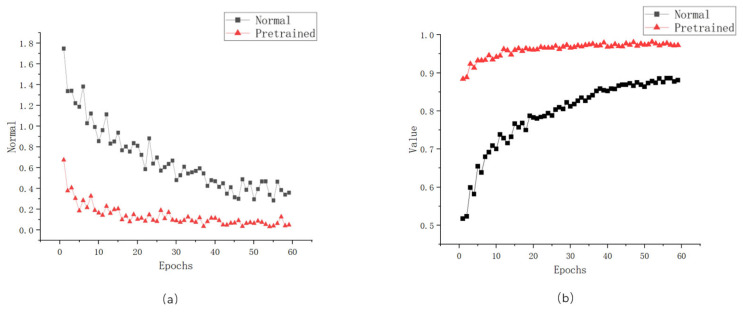
Comparison of the effects of pretrained models on model training: (**a**) loss value figure; (**b**) accurate figure.

Taking MobileNet_v3_Small as an example, the comparison of pretrained models is illustrated in Figure 10. The loss values of the models using pretraining converged more quickly than those of the normally trained models (as shown in Figure 10a), reaching a desirable convergence state in the early stages of training. Consequently, under the same number of training epochs, models trained with pretraining exhibited higher performance. Furthermore, the accuracy (Acc) of the models using pretrained models maintained a higher precision compared to those without pretraining, achieving high performance from the early stages of training (as depicted in Figure 10b).

As shown in Table 1, the final accuracies of the three models that underwent pretraining through transfer learning all showed improvements, with increases of 12.40%, 7.35%, and 9.5%, respectively. Therefore, it can be concluded that pretrained models played a significant role in enhancing the performance of the models. Moreover, with the demonstration of the superiority of the DTSD technique, all subsequent experiments utilized transfer learning for pretraining.

### 3.4. Improved Model Verification

The above experiments provide evidence that the use of a pretrained model contributes to an improvement in the performance of the model. To further verify the effect of the DTSD model on MobileNet_v3_Small, the high-precision teacher models ResNet50_vd (98.9%) and DenseNet121 (98.7%) were trained on the 10 Chinese herbal medicine augmented datasets in advance.

In order to obtain optimal performance for MobileNet_v3_Small_DTSD, three learning rate decline strategies were implemented while applying the improved knowledge distillation DTSD. These strategies were exponential warmup, piecewise and cosine. When the other experimental parameters were the same, the DTSD distillation model using the exponential warmup learning rate decline strategy had the highest accuracy of 98.60%. Consequently, as shown in Table 2, it can be inferred that the learning rate decline strategy of exponential warmup conferred advantages to the DTSD training model.

**Figure 11:**
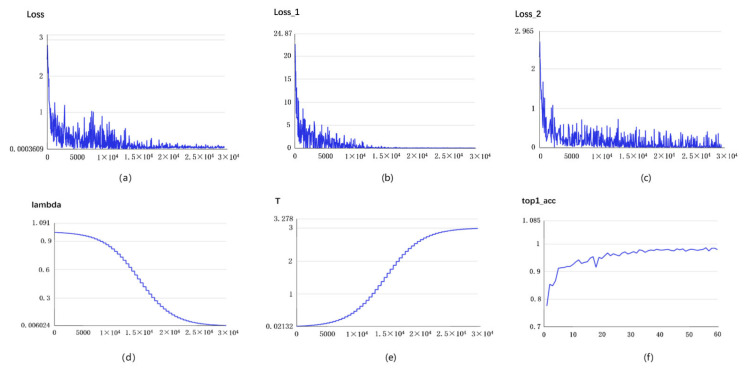
Metrics of mobileNet_v3_Small_DTSD changes in training process:(**a**) total loss; (**b**) soft loss; (**c**) hard loss; (**d**) λ value change; (**e**) T value change; (**f**) accuracy change curve.

The parameter and metric changes of the whole training process of MobileNet_v3_Small_DTSD are shown in Figure 11, where variables 11a, 11b, and 11c denote the changes in total loss, soft loss and hard loss, respectively. The total loss is the overall loss of the model during the training process, which is usually a combination of multiple loss functions. When training a neural network, there are usually multiple tasks or multiple loss metrics, and each loss function corresponds to one task or one metric. The total loss is the weighted sum or average of these loss functions and is used to measure the performance of the entire model. Soft loss is a technique used in training, mainly to help the model learn better. Soft loss is usually achieved by introducing some extra penalty or regularization terms in the loss function, which can help the model generalize better to new data and avoid overfitting. The introduction of a soft loss can help to adjust the learning direction of the model to better fit the training data. Hard loss usually refers to the loss calculated in the inference stage of the model, which is the performance of the model on the test data. Hard loss is the difference between the true label and the predicted label of the model, which is used to measure the prediction accuracy or performance of the model. During training, the model usually adjusts its own parameters by optimizing the total loss to minimize the hard loss. It is evident from the figure that the model achieved satisfactory convergence during the initial phase of training. In Figure 11d, the *λ* value (lambda) is usually used in regularization terms (e.g., L1 regularization, L2 regularization) to balance the model’s fitting effect with the effect of the regularization terms. The *λ*-value change curve shows the effect of different *λ* values on the model’s performance during the training process. λ decays in an inverted S-shape, which means that the student model mainly performs distribution fitting with the hard label through transfer learning in the early stage, and gradually shifts the learning center to the soft label of the teacher model with the deepening of the experiment. The *T* value (temperature) is usually used in temperature-regulated soft label methods to smooth the label distribution to improve the training of the model. The *T*-value variation curve shows the effect of different *T* values on the performance of the model during the training process. However, the temperature *T* depicted in Figure 11e exhibits a contrasting situation. During the initial phase, the correct label part of the teacher model is mainly enlarged, so that the student model can quickly fit. In the subsequent phase, the proportion of dark knowledge is gradually expanded, because the accuracy of the student model can be further enhanced. The accuracy change curve shows how the accuracy of the model changes during the training process. The accuracy on the training set and the validation set are usually treated as two separate parts of the curve. This curve can be used to observe whether the model is overfitting or underfitting. Figure 11f reflects the evaluation accuracy of MobileNet_v3_Small_DTSD. Once the model quickly reaches good performance in the early stage, it mainly focuses on acquiring the dark knowledge from the teacher model to achieve higher accuracy in the subsequent stage.

### 3.5. Comparative Experiments with Similar Models

To prove the superiority of the best MobileNet_v3_Small_DTSD model, which was achieved by the adjustment of the learning rate decline strategy, a comparative analysis was conducted with MobileNet_v3_Small using various techniques. The comparison models were mainly as follows: (1) MobileNet_v3_Small without a transfer learning pretrained model; (2) MobileNet_v3_Small_Pre with pretrained models; (3) MobileNet_v3_Small_SSLD trained with SSLD (semi-supervised label knowledge distillation) technique.

According to the data presented in Table 3, it can be observed that the improved DTSD technique achieved an accuracy of 98.60% under identical training parameters. This accuracy was notably 11.15% higher than that of the original training model. Furthermore, the improved DTSD technique outperformed the SSLD technique, which also incorporates knowledge distillation, by 1.50%. Furthermore, the DTSD technique surpasses the PRE model, which uses transfer learning, by 1.35%.

**Figure 12:**
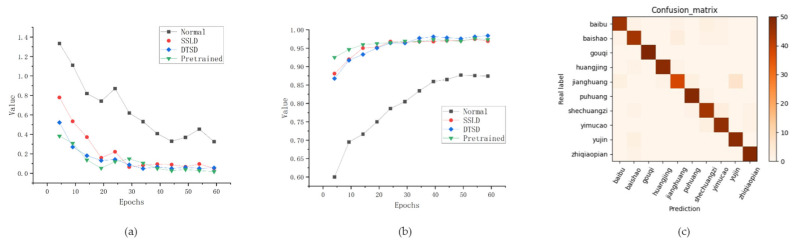
Performance comparison of MobileNet series: (**a**) loss; (**b**) Accuracy; (**c**) Confusion matrix.

As shown in Figure 12, the normally trained model in Figure 12a performed poorly in terms of both aspects (loss and accuracy), while the improved DTSD model maintained the same convergence state as the other two models PRE and SSLD in terms of loss. In Figure 12b, the circular green line (using the DTSD technique) is compared with the square red line (using transfer learning). During the initial stage of training, the performance of the DTSD model is inferior to that of the transfer learning model. The ongoing decay of *λ* causes the student model to shift the training focus to the soft label of the teacher model, and the constant growth of temperature *T* expands the proportion of dark knowledge, which makes the student model learn more parameters. These two factors make an obvious intersection point appear in the two graphs. Furthermore, DTSD successfully achieved a leadership position after coming from behind. The confusion matrix diagram in Figure 12c is the performance of MobileNet_v3_Small_DTSD on the test dataset under the training parameters. Eventually, the model performed well. Therefore, the utilization of the DTSD technique inside the same model should help to break through the limitation of the model accuracy.

### 3.6. Experimental Comparisons with Other Models

To further verify the performance of the DTSD technique, it was compared with other mainstream models including EfficientNetB1, Xception65, ResNet50_vd, DenseNet121, and others. The main evaluation criteria encompassed accuracy, model volume, and prediction cost, where the prediction cost was the average time of predicting 500 test herbal images. DenseNet121 is a model in the DenseNet family. Dense connections in DenseNet help alleviate gradient sparsity, making the model easier to train and improving its generalization ability. Dense connections allow features to be passed through shorter paths, increasing the efficiency of information transfer and reducing information loss. ResNet50_vd is relatively deep, with 50 layers, which allows the model to learn higher-level abstract features and improve the ability to capture and represent complex patterns. Xception65 utilizes a structure of depth-separable convolution, which divides standard convolution into two steps: deep convolution and point-by-point convolution. This structure helps to reduce the number of parameters and improve the computational efficiency of the model.

EfficientNetB1 employs a compound coefficient (composite coefficient) approach to design an efficient model structure by scaling the depth, width, and resolution of the network in a balanced way. This structure can achieve better performance with certain computational resources.

The training parameter values for the experiments in this paper are set as shown in Table 4, Batch size is 16, Basic learning rate is 0.0037, Learning rate decline strategy used is ExponentialWarmup, Epoches is 60, and pre-training of the model is performed.

As shown in Table 5, when comparing different models horizontally, it is evident that MobileNet_v3_Small, which uses DTSD technology for knowledge distillation, achieved an accuracy of 98.60%, ranking second in terms of accuracy. However, its model size (10 MB) and prediction cost (0.0172 s) were optimal among the five models. The feasibility of the DTSD technique was thus proved.

### 3.7. Application

**Figure 13:**
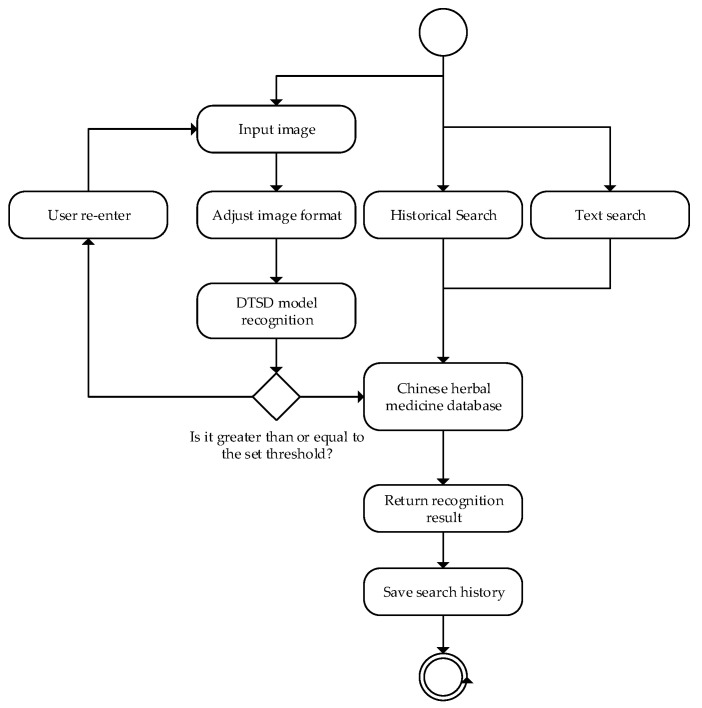
Process design of our Chinese herbal medicine identification system.

**Figure 14:**
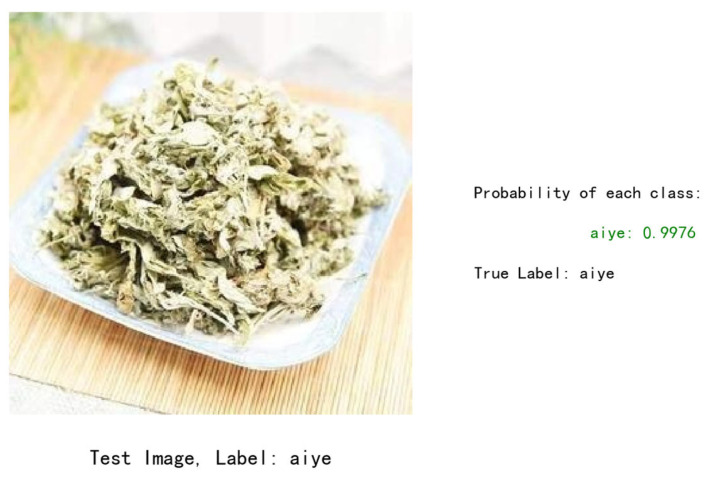
The system outputs the result of the test image.

To validate the practical application of the model, we developed a mobile recognition app based on it. As shown in Figure 13, the app’s main features include an input image, a historical search, and text search capabilities. After selecting the image search function, users can upload relevant images of Chinese herbs. The app processes these images through cropping and utilizes the corresponding model and a backend database of Chinese herbs to return information about the herb. The example result, shown in Figure 14, indicates that the tested image has been classified as “aiye” with a matching true label. The probability of correct classification is 99.76%. Practical validation confirmed that the MobileNet_v3_DTSD model, trained using the dual-teacher adaptive-decay approach based on improved knowledge distillation and data augmentation, maintained its lightweight and rapid processing characteristics while also exhibiting robustness and high accuracy. Future efforts will focus on further optimizing these improvements to enhance the model’s performance in real-life applications.

## 4. Conclusions

In this paper, to realize lightweight Chinese herbal medicine image recognition on a mobile terminal, a Chinese herbal medicine recognition model with dual-teacher supervised decay based on knowledge distillation was proposed. By improving the single-teacher model to a dual-teacher model, the output soft label, adaptive decay parameters, and dynamic combination loss of the teacher model, it was applied to the lightweight model network MobileNet_v3, and finally deployed into a mobile application. The experimental results indicated that the mean classification accuracy of a set of 10 Chinese herbal medicine images was 98.60%. Moreover, the average time taken to identify a single image was 0.0172 s, and the model size was 10 MB. Upon successful deployment of the application, it demonstrated the capability to fulfill the speed and accuracy requirements of real-life scenarios, hence offering valuable technical reference for mobile phone applications. Despite the remarkable results achieved in this paper in lightweight herbal image recognition, there are still some shortcomings that need further consideration and improvement. The robustness of the model in dealing with real-world challenges such as complex scenes and lighting changes still needs to be improved to ensure high accuracy in a variety of environments. The next step is to expand and diversify the dataset to improve the model’s adaptability to different herbal species and environmental conditions. Secondly, techniques such as adversarial training should be introduced to enhance the robustness of the model against noise and interference.

## Figures and Tables

**Table 1:** Accuracy of the models with of pretraining.

Network	Pretraining Model	Accuracy (%)
ResNet50_vd	×	86.10
	√	98.50
DenseNet121	×	91.05
	√	98.40
MobileNet_v3_Small	×	88.35
	√	97.85

“×” indicates that the model has not been pre-trained. “√ “ indicates that the model has been pre-trained.

**Table 2:** Comparison of parameter combination results of MobileNet_v3_Small_DTSD.

Student Model	Teacher Model	Acc Of Teacher Model (%)	Learning Rate Decline Strategy	Accuracy (%)
MobileNet_v3_Small	ResNet50_vd	98.90	Piecewise	97.80
	DensNet121	98.70		
MobileNet_v3_Small	ReNet50_vd	98.90	Cosine	98.15
	DenseNet121	98.70		
MobileNet_v3_Small	ResNet50_vd	98.90	Exponential warmup	98.60
	DenseNet121	98.70		

**Table 3:** Comparison results with MobileNet_v3_Small.

Learning Rate Decline Strategy	Batch Size	Accuracy
MobileNet_v3_Small	16	86.45
MobileNet_v3_Small_PRE	16	97.25
MobileNet_v3_Small_SSLD	16	97.10
MobileNet_v3_Small_DTSD	16	98.60

**Table 4:** Setting of training parameters.

Parameter	Batch Size	Basic Learning Rate	Learning Rate Decline Strategy	Epochs	Pretraining Model
Parameter value	16	0.0037	Warmup	60	√

“√ “ indicates that the model has been pre-trained.

**Table 5:** Comparison of results between MobileNet_v3_Small_DTSD and other mainstream models.

Network	Accuracy (%)	Model Volume (MB)	Prediction Time (s)
DenseNet121	97.10	29.30	0.0186
ResNet50_vd	97.35	90.90	0.0237
Xception65	97.65	131	0.0198
EfficientNetB1	98.95	27.5	0.0233
MobileNet_v3_Small_DTSD	98.60	10	0.0172

## Data Availability

The data presented in this study are available on request from the corresponding author.
